# COVID-19 vaccination uptake in remote areas—Evidence from a panel survey in Bangladesh

**DOI:** 10.1371/journal.pone.0305659

**Published:** 2024-08-09

**Authors:** Lukas Rudolph, Vally Koubi, Jan Freihardt

**Affiliations:** 1 Department of Politics and Public Administration, University of Konstanz, Konstanz, Germany; 2 Center for Comparative and International Studies, ETH Zurich, Zurich, Switzerland; National Institute of Preventive and Social Medicine, BANGLADESH

## Abstract

**Background:**

Vaccination has proven to be an essential strategy in combating the COVID-19 pandemic. This study aims to discern the factors influencing both the intentions for and actual behavior regarding COVID-19 vaccination among remote, rural populations in Bangladesh.

**Methods:**

The study utilized panel survey data comprising 1,698 randomly selected household heads. These are predominantly illiterate, of Muslim religion, middle-aged, and male, with agriculture or day labor as primary income source. They reside in 36 locations distributed along the whole 250 km length of the Jamuna River in Bangladesh. Data collection occurred through face-to-face and telephone interviews conducted between September 2021 and October 2022. Descriptive statistics and Ordinary Least Squares regression models were employed to assess influence factors for COVID-19 vaccination intentions and uptake. The analyses considered the constructs of the Health Belief Model alongside sociodemographic characteristics such as gender, age, religion, education, and income source.

**Results:**

Survey respondents showed a notably high willingness to receive the COVID-19 vaccine promptly upon its availability. However, the effectiveness of the Health Belief Model in elucidating COVID-19 vaccination uptake was limited, except for its availability component. Older individuals, those with higher levels of education, and individuals employed in government or formal sector occupations were prompt in receiving the COVID-19 vaccine as it became available. Gender, religion, and the presence of dependents in the household did not exert a significant influence on vaccination uptake.

**Conclusions:**

The results indicate that a strong willingness to receive the COVID-19 vaccine correlated with an increased likelihood of vaccine uptake once it was available. These findings suggest that a widespread distribution of COVID-19 vaccines to low-income and remote areas could have served as a vital strategy in mitigating the COVID-19 pandemic.

## Introduction

Vaccination is one of the most efficient ways to prevent and control infectious diseases and save lives [1], and vaccines against COVID-19 were instrumental in fighting the pandemic by reducing the probability of severe illness and death [2]. Yet, COVID-19 vaccine uptake rates have varied across countries and different communities and ethnic groups [3, 4]. Moreover, it has been shown that the local effectiveness of immunization campaigns depends on the supply side, e.g., staff availability and equipment [5] and vaccine distribution [6], as well as on demand-side factors, e.g., public acceptance [7]. In this study, we direct our attention to the latter in the context of the global COVID-19 pandemic, focusing specifically on Bangladesh, a country situated in the Global South. Furthermore, our study targets remote, rural communities dwelling along the Jamuna River, an overlooked population primarily due to the challenges associated with reaching and surveying these communities (for notable exceptions see [8, 9]). These communities face heightened vulnerability to natural hazards like river flooding and riverbank erosion, alongside a low level of development marked by limited human capital (such as low literacy rates), financial constraints (high poverty rate), and inadequate physical infrastructure (including poor housing conditions and limited land ownership).

**Fig 1:**
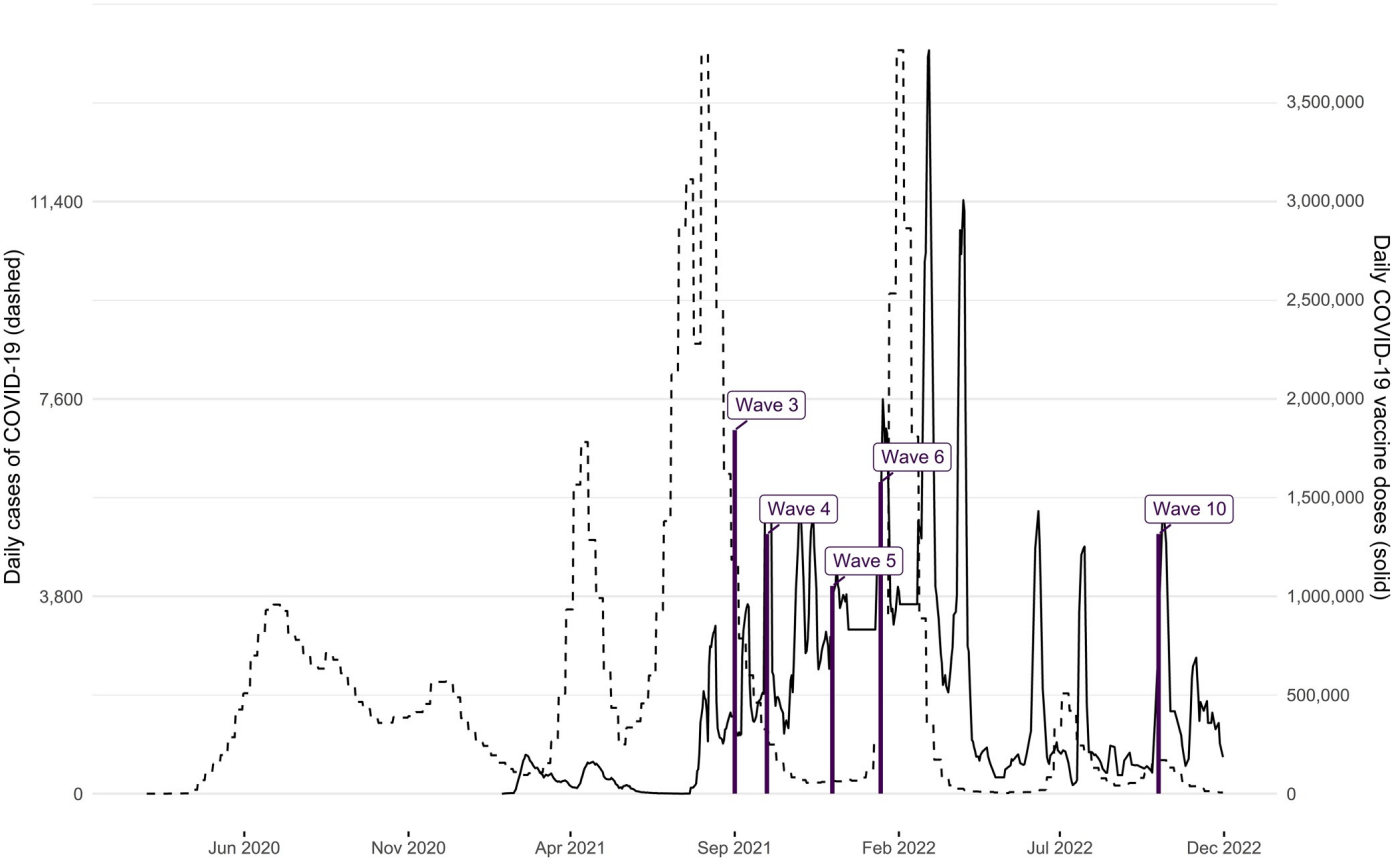
Dashed line: Weekly cases of COVID-19 in Bangladesh, March 2020—November 2022. Solid line: Daily COVID-19 vaccine doses administered in Bangladesh, January 2021—November 2022. Source: Our World in Data [34]. All doses, including boosters, are counted individually. Spikes denote the timing of survey waves.

The onset of COVID-19 cases in Bangladesh was confirmed on March 8, 2020. Subsequently, there was a steady increase in infections and deaths, peaking in early spring and summer 2021 and again in the early months of 2022 (see [10] and Fig 1, dashed line, depicting the weekly number of infections from March 2020 to November 2022.) Bangladesh initiated its vaccination campaign, starting with frontline health workers, on January 27, 2021, followed by the general public on February 7, 2021. Initially, only the Oxford-AstraZeneca vaccine was authorized for emergency use from January to April 2021. However, due to a shortage of vaccines, Bangladesh approved the emergency use of two additional vaccines: the Russian Sputnik V and China’s Sinopharm BIBP. In May/June 2021, the government approved the Pfizer-BioNTech and Moderna COVID-19 vaccines, intended for distribution through the COVAX initiative. By October 2021, a total of seven COVID-19 vaccines had received full approval in Bangladesh. To facilitate vaccination, the government launched a web portal (called Surokkha), allowing citizens to register for vaccination using their National Identity Card (NID) details. Initially limited to individuals aged 55 and above, the eligibility criteria were gradually expanded to include those aged 40 and above, and later lowered to 30 years in July 2021 due to lower-than-expected take-up. The vaccination eligibility was extended to university students in September 2021, followed by the 12 to 17-year-olds in school in November 2021 [11, 12]. Overall, this was reflected in increasing vaccination administration in Bangladesh, beginning in September 2021 and peaking in early 2022 (see Fig 1, solid line, for daily COVID-19 vaccine doses administered from January 2021 to November 2022.).

Regarding research on COVID-19 vaccination, a seminal study offers an initial comprehensive insight indicating that vaccine acceptance rates were surprisingly high in low- and middle-income countries during late 2020 and early 2021 [13]. This finding aligns with findings within the context of our country study, Bangladesh, where the majority of respondents in various surveys, ranging from 85% [14] to 60% [15] expressed a willingness to receive the vaccination during the specified time frame (see also [16–18]). Although all of these studies report high COVID-19 vaccination intentions, these intentions exhibit variations contingent upon sociodemographic characteristics. For instance, [17] found that disadvantaged and remote groups such as the elderly, low-income individuals, those with low levels of education, people with chronic illnesses, and residents of rural or slum areas, as well as certain occupations like day laborers and farmers, expressed lower vaccination intentions (see also [19, 20]). Other studies report that fear of side effects associated with the vaccine [15] and concerns about whether the vaccine was “halal” (i.e., permissive for consumption according to Islamic rules) [21] impeded respondents’ willingness towards getting vaccinated, or that respondents were skeptical of vaccine effectiveness [18]. Underlying reasons for these differences in sociodemographics likely stem from a combination of accessibility issues, lack of information, prevailing norms, and personal attitudes [22]. Overall, these studies suggest that the primary challenge to vaccination uptake was not a lack of willingness among surveyed populations, but rather that issues related to information about vaccine availability, effectiveness and outreach of vaccination campaigns, and ease of the registration process prohibited vaccinations [23]. This was particularly the case in rural areas, where limited digital literacy and access to online registration forms posed significant challenges for the average citizen, only eased by changes in the vaccination program such as walk-in registration. Nevertheless, once respondents successfully completed the registration process, they self-reported high vaccination uptake [23].

Yet, we still lack empirical evidence across three dimensions: Firstly, the determinants of vaccination intentions among remote and hard-to-reach populations remain inadequately elucidated; secondly, the translation of these intentions into actual behavior warrants further investigation; and third, there is a need to explore how intentions and behavior evolve over time for different sociodemographic subgroups.

In this article, we explore COVID-19 vaccination intentions and crucially extend our analysis beyond mere intentions to investigate actual behavior seven to twenty months after the onset of the vaccine rollout in Bangladesh. Our theoretical underpinnings are grounded in the Health Belief Model (HBM). The theoretical framework of the HBM has been extensively utilized in research examining vaccination intentions and uptake across various communicable diseases, including influenza [24], swine flu, and hepatitis B [25]. Recent literature highlights the significance of the HBM’s theoretical constructs as predictors of COVID-19 vaccination intentions [26–31]. In a nutshell, the HBM seeks to explain and predict preventive health behavior in terms of certain belief patterns. It posits that there are different negative and positive factors influencing vaccination intentions and behavior as well as the adoption of healthy behavior in general. These factors include perceived susceptibility, perceived severity of the health risk, perceived benefits, and perceived barriers [25, 28, 32]. We then contrast the HBM perspective with sociodemographic factors such as age, gender, education, profession, and income source, which have been found to directly influence health behavior decisions.

## Materials and methods

### Study design, setting, and participants

In our survey design, meticulous attention was devoted to constructing a high-quality panel that would facilitate the generalization of our findings to remote, rural populations. Our empirical analysis is based on a pre-registered sampling frame for a panel survey encompassing 1,698 household heads, defined as the primary decision-makers within their households, residing in 36 locations distributed along the whole 250 km of the Jamuna River in Bangladesh. These household heads are participants in a large-scale, multi-year survey project initiated in March 2021, and were selected according to a multi-stage clustered design: in the first stage of the sampling procedure, 250 potential survey locations, delineated as one-kilometer stretches along the easternmost riverbank line of the Jamuna River were identified. Subsequently, after excluding stretches with an insufficient number of settlements defined as fewer than 75 houses, 36 locations were selected for inclusion in our sample. Within each of these selected 36 locations, households were sampled utilizing a stratified random spatial sampling design. Specifically, within each location, a set of 75 random latitude-longitude points were generated using ArcMap software ensuring a minimum distance of 10m between points [33]. In the field, enumerators navigated to these designated points using smartphones. Upon reaching each point, enumerators identified the house closest to the specified point based on visual estimation and subsequently proceeded to interview the household head. In instances where the household head declined participation or was unavailable after two contact attempts, enumerators moved to the next closest household, in relation to the starting point. Consequently, we are confident that our respondents constitute a high-quality representative sample of the rural population residing in this region of Bangladesh. An overview of the 36 locations is provided in Fig 1 in S1 Appendix. Following the establishment of this initial sample, and given that we collected respondents’ phone numbers, we followed up with this sample using high-frequency phone interviews every six to eight weeks.

Regarding the underlying population we study, we have no up-to-date census data at a fine-grained level. However, given the random spatial draw utilized for sampling, our survey statistics offer valuable insights into the characteristics of the broader population under study. Table 1 in the Results section presents corresponding information (as of December 2021). As can be seen from the sociodemographic indicators situated at the top of Table 1, we observed mostly an uneducated population, approximately three-quarters are characterized as illiterate or able only to sign their name, and only slightly less than a quarter have at least primary or secondary school education; which predominantly works in agriculture (27%) or day labor (31%); and with the vast majority identifying as adherents of the Muslim faith (95%). Additionally, a substantial number of households do not support old-aged members under their roof (65%), while a majority provide support for children under 15 years (72%). Household heads are predominantly males (87%), with a notable proportion falling within the age brackets of 30–44 (35%) and 45–64 years (42%).

**Table 1:** Descriptives for survey respondents as of December 2021.

	Statistic
Categorical variables	N (%)
Age	
Below 30	132 (7.79)
30–44	601 (35.46)
45–64	713 (42.06)
65 and over	249 (14.69)
Main income source	
Agriculture	458 (27.41)
Own business	319 (19.09)
Employed	110 (6.58)
Day labor	514 (30.76)
Government job or similar	69 (4.13)
Other	201 (12.03)
Gender	
Male	1480 (87.16)
Female	218 (12.84)
Education	
Illiterate	1030 (60.7)
Signature only	267 (15.73)
Primary school	203 (11.96)
Secondary school and above	197 (11.61)
Religion	
Hinduism	28 (1.65)
Islam	1669 (98.35)
Household members under 15	
None	474 (27.93)
1	480 (28.29)
2	474 (27.93)
3	189 (11.14)
4+	80 (4.71)
Household members above 65	
No	1418 (83.56)
Yes	279 (16.44)
Vaccine availability (stated)	
No	1273 (84.53)
Yes	233 (15.47)
Received at least one vaccination	
No	278 (18.4)
Yes	1233 (81.6)
Personal life was affected by COVID	
No	1161 (79.68)
Yes	296 (20.32)
Susceptibility of the population to COVID	
Rather the sinful	1147 (80.95)
Same/rather the devout	270 (19.05)
Rather the rich	1061 (72.37)
Same/rather the poor	405 (27.63)
**Continuous variables (range)**	**Mean [Median (IQR)]**
COVID is a threat to family family (1–5)	2.01 [2 (0)]
Satisfaction with personal health (1–5)	3.79 [4 (0)]
Pandemic will end within 6 months (1–4)	2.27 [2 (1)]
New wave expected within 6 months (1–4)	2.35 [2 (1)]
Perceived vaccination rate in the village (0–5)	3.31 [4 (1)]
Perceived affectedness in the village (0–5)	0.32 [0 (0)]
Travel duration to district capital (hrs.)	2.11 [1.64 (2.33)]
Observations	1698

Summary statistics for respondents (from wave 5 data). N differs by variable because of item non-response.

### Data collection procedure

The survey data used in this study was collected across consecutive survey waves through varying modes, including telephone interviews in September, October, and December 2021, as well as in October 2022, and face-to-face interviews in January/February 2022, all conducted in Bangla by native enumerators. The timing of the survey waves, relative to the development of the COVID-19 caseload and vaccination roll-out, is presented in Fig 1.

The co-authors collaborated with a team of Bangladeshi enumerators, whom they recruited primarily from Bangladeshi universities, and subsequently provided training and supervision throughout the fieldwork phase. Enumerators underwent extensive, multi-day training sessions facilitated by the co-authors. These were conducted in person for the in-person survey waves and via video conferencing for the telephone surveys. Enumerators conducting telephone surveys were from among the pool of enumerators who also were trained to conduct the in-person interviews. Data was collected by enumerators using the Qualtrics Offline Survey App. The co-authors oversaw the data collection effort, checked incoming data for errors, and instructed enumerators to correct these on a daily basis. Respondents received a modest monetary incentive as a token of appreciation for their participation in the survey. Attrition rates between survey waves remained remarkably low, ranging from 6% at wave 6 to 22% at wave 10, relative to the baseline survey wave 1. The primary reasons for attrition were attributed to non-availability stemming from difficulties in reaching respondents via phone, or limited time availability on the side of respondents. Consequently, the majority of respondents were successfully re-interviewed following one or two missed survey waves, with only a negligible proportion permanently discontinuing their panel participation.

### Data collection instrument

The questionnaires employed in this study included both closed and open-ended questions pertaining to respondents’ attitudes and behavior towards COVID-19 vaccination as well as personal and household information. Specifically, the survey instruments solicited information pertaining to respondents’ age, gender, religion, marital status, educational attainment, primary source of income, monthly household income, household size, and number of children and older individuals. Furthermore, respondents were also asked additional questions regarding their perceptions of COVID-19 as a health threat to themselves and their families, the perceived severity of the pandemic, and their awareness of the availability of the COVID-19 vaccines. Summary statistics for these variables are presented in Table 1. Note that our questionnaires were pre-registered. Links to the full questionnaires and the exact wording of core questionnaire components related to COVID-19 are compiled in section 3 of S1 Appendix.

### Ethics statement

Ethical clearance for this study was secured from ETH Zurich Ethics Commission under decision EK 2020-N-67. Prior to participation in the surveys, respondents provided verbal consent for the telephone interviews and written consent for the in-person interviews (for details, see section 2 of S1 Appendix).

### Variables and measurement

The following provides an overview of our study’s core independent and dependent variables, including their measurement. For exact question wording, see section 3 of S1 Appendix.

#### Independent variables

Regarding our independent variables, a first set comprises sociodemographic variables. These sociodemographic variables encompass gender (female or male), age groups (younger than 30, 30 to 44, 45 to 64, or older than 64 years), educational attainment (illiterate, possessing only the ability to provide a signature, having primary education, or having secondary education), the primary source of income (including categories such as agriculture, business ownership, employment in the private sector, employment in the government sector, day laborer, or other), religious affiliation (Muslim or Hindu), and household dependents (qualified by the number of children under 18 years of age and elderly individuals aged above 65 years residing in the household).

A second set comprises components of the Health Belief Model (HBM). In this study, we investigate the constructs of the HBM as follows: *Perceived benefits*, referred to as health motivation, is operationalized through respondents’ perceptions regarding the beneficiaries of their vaccine uptake, categorized as either themselves, their family members, or their community. *Perceived severity* is approximated through questions gauging respondents’ expectations concerning the imminent cessation of the pandemic and the anticipation of a subsequent wave occurring soon. *Perceived susceptibility* is proxied through four inquiries: respondents’ assessment of COVID-19 as a personal threat, their perception of personal health satisfaction, their prior experience with COVID-19, and their identification of societal groups deemed most vulnerable to COVID-19 (including distinctions such as urban versus rural, sinful versus devout, rich versus poor, and young versus old).*Perceived barriers*, referred to as availability is operationalized by measuring travel duration to the nearest district capital (indicative of remoteness), and by an indicator capturing respondents’ assertion of vaccine unavailability. Finally, recognizing the significant impact of social context on individual intentions and behaviors, a variable reflecting *social cues* is incorporated, capturing respondents’ perceptions concerning village-level COVID-19 vaccination efforts and the prevalence of COVID-19 within their village [35]. These constructs are assessed with data from survey wave 5, conducted in December 2021, as this wave encompasses the HBM indicators delineated above.

#### Dependent variables

Our core dependent variable is *vaccination status*, which was evaluated through self-reporting of the number of vaccine shots received by the respondent at each survey wave, ranging from zero to four (inclusive of up to two booster shots). To enhance interpretability and analytical flexibility, this variable was re-coded to several binary indicators of having received at least one shot in September 2021/October 2021/December 2021/January 2022 or at least one booster shot by October 2022, and an indicator of being fully vaccinated (two shots) in December 2021. For respondents who had not yet received any vaccine shot, their *vaccination intentions* were assessed. This was gauged by the question “Would you accept a COVID-19 vaccination for yourself if it became available?” followed up with a binary yes/no scale.

### Statistical analysis

Initially, we present descriptive statistics for a broad set of indicators pertaining to vaccination intentions, vaccination perceptions, and policy preferences among our survey participants. These statistics are presented in the form of frequencies and percentages. Subsequently, we employ linear regression models to explore two aspects: a) the relationship between the HBM components (see above) and COVID-19 vaccine uptake, and b) the association between sociodemographic variables and COVID-19 vaccine uptake. To ensure the reliability of the findings, we conducted checks for multicollinearity among regressors, with results indicating minimal concerns, as reported in the notes to Tables 2 and 3. We use linear probability models for the core impact regressions, as these can be easily interpreted and regularly show similar effects compared to logistic regressions [36]. Nonetheless, acknowledging that the technical assumptions of linear regression might not be met given the binary nature of our outcomes, we provide supplementary logistic regression models in Tables 1 and 2 in S1 Appendix. We report robust standard errors, or alternatively standard errors clustered by respondent location (i.e., by village). Moreover, we use fixed effects for village location in all models as a preferred specification—as these allow to control for all constant location-specific factors. Given that locality is a core determinant of the outcome variable—partially, location predicts vaccination reception or no reception perfectly for some survey waves and outcomes –, we also report logistic regression results without location fixed effects as a robustness test in Tables 1 and 2 in S1 Appendix. Finally, regarding the sociodemographic variables, we leveraged the panel property of our dataset to take a dynamic perspective. For this, we drew on a random effects model, specifically tracing vaccination rates within key subgroups defined by age, occupation, religion, and education. To accomplish this, we regressed the absolute number of vaccine shots received ranging from zero to four, including the second booster shot, on the aforementioned sociodemographic factors, time-fixed effects, and their interaction, as well as location-fixed effects. This approach enabled us to generate linear predictions for the average number of shots received within the respective sociodemographic subgroup, while simultaneously controlling for other relevant factors in the analysis. All analyses were performed using the statistical software Stata 16. The data was cleaned and merged across survey waves in the statistical software R by the authors.

**Table 2:** Health belief model for vaccination uptake.

	(1)	(2)	(3)	(4)
Dependent variable: Received vaccination shot, at least:	one as of Dec. ’21	two as of Dec. ’21	one as of Dec. ’21	two as of Dec. ’21
**Health motivation**				
Covid threat to family health	0.01	-0.02	0.01	-0.01
	(0.01)	(0.02)	(0.02)	(0.02)
Personal health satisfaction	0.00	0.03	0.04*	0.06*
	(0.01)	(0.02)	(0.02)	(0.02)
Personal Covid affectedness = 1	0.02	0.03	0.05	0.06
	(0.02)	(0.04)	(0.04)	(0.04)
**Severity**				
Pandemic ends within 6 months	0.00	-0.00	0.01	0.00
	(0.01)	(0.02)	(0.02)	(0.02)
New wave expected within 6 months	-0.01	-0.02	-0.03	-0.04^+^
	(0.01)	(0.02)	(0.02)	(0.02)
**Susceptibility**				
Rather the sinful	0.01	-0.01	-0.01	-0.02
	(0.01)	(0.05)	(0.04)	(0.06)
Rather the rich	-0.00	-0.06	0.03	-0.03
	(0.02)	(0.05)	(0.04)	(0.05)
**Social cues**				
Perceived vaccination rate in village	-0.01	-0.01	0.03^+^	0.01
	(0.00)	(0.02)	(0.01)	(0.02)
Perceived affectedness in village	-0.00	0.03	-0.01	0.02
	(0.01)	(0.02)	(0.02)	(0.03)
**Availability**				
Travel duration most prox. district capital (hrs.)	-0.03**	-0.43**		
	(0.01)	(0.01)		
Vaccine not available (stated)	-0.97**	-0.63**		
	(0.01)	(0.05)		
Constant	1.06**	1.80**	0.64**	0.54**
	(0.06)	(0.15)	(0.10)	(0.14)
Location fixed effects	Yes	Yes	Yes	Yes
*N*	827	827	828	828
*R* ^2^	0.86	0.37	0.14	0.18

OLS regression of the dependent variable indicating vaccination take-up (see model header: binary indicator of at least one dose in models 1 and 3; of two doses, i.e., fully vaccinated, in models 2 and 4) on regressors derived from the HBM. Location-fixed effects are included. Robust standard errors are presented in parentheses. The centered variance-inflation factor is at 1.77 (models 3/4) and 3.60 (models 1/2) on average, with the ‘travel duration’ indicator being the sole variable to exceed the cut-off of 5 for problematic values due to its correlation with the village-fixed effects.

^+^ (*p* < 0.10),

* (*p* < 0.05),

** (*p* < 0.01),

*** (*p* < 0.001).

**Table 3:** Sociodemographic characteristics and vaccine take-up.

	(1)	(2)	(3)	(4)	(5)
Dependent variable: Received vaccination shot, at least:	one as of Sept. ’21	one as of Oct. ’21	one as of Dec. ’21	one as of Jan. ’22	three as of Oct. ’22
**Age of household head**					
<30	(ref.)	(ref.)	(ref.)	(ref.)	(ref.)
30–44	0.02	0.03	0.08*	0.06*	0.14*
	(0.05)	(0.05)	(0.04)	(0.03)	(0.06)
45–64	0.05	0.05	0.08^+^	0.06*	0.15*
	(0.04)	(0.04)	(0.04)	(0.03)	(0.06)
65+	0.07	0.10^+^	0.09^+^	0.02	0.16^+^
	(0.05)	(0.05)	(0.05)	(0.05)	(0.08)
**Main income source of household**					
agriculture	(ref.)	(ref.)	(ref.)	(ref.)	(ref.)
own business	0.03	0.07*	-0.03	0.04^+^	-0.02
	(0.03)	(0.04)	(0.03)	(0.02)	(0.05)
employed	0.09*	0.03	-0.00	0.02	0.03
	(0.04)	(0.05)	(0.05)	(0.03)	(0.05)
daylabor	-0.02	0.03	-0.01	-0.01	-0.02
	(0.02)	(0.03)	(0.03)	(0.02)	(0.04)
government or comparable	0.31***	0.29***	0.16***	0.13***	0.07
	(0.06)	(0.06)	(0.04)	(0.03)	(0.04)
other	0.04	0.11*	0.01	0.03	0.08^+^
	(0.03)	(0.04)	(0.04)	(0.02)	(0.05)
**Gender of household head**					
male	(ref.)	(ref.)	(ref.)	(ref.)	(ref.)
female	-0.03	0.04	0.01	0.01	0.08
	(0.03)	(0.04)	(0.04)	(0.02)	(0.05)
**Education level of household head**					
illiterate	(ref.)	(ref.)	(ref.)	(ref.)	(ref.)
signature only	-0.03	0.03	-0.01	-0.02	-0.01
	(0.02)	(0.03)	(0.03)	(0.03)	(0.05)
primary education	0.03	0.03	-0.04	-0.05*	-0.00
	(0.03)	(0.05)	(0.04)	(0.02)	(0.04)
secondary education or above	0.14***	0.08^+^	-0.00	-0.06*	0.04
	(0.04)	(0.05)	(0.04)	(0.03)	(0.05)
**Religion of household head**					
Hindu	(ref.)	(ref.)	(ref.)	(ref.)	(ref.)
Muslim	-0.08	-0.05	-0.06	-0.02	-0.30**
	(0.06)	(0.14)	(0.08)	(0.04)	(0.09)
**Children in household**					
none	(ref.)	(ref.)	(ref.)	(ref.)	(ref.)
1	0.04^+^	0.00	-0.02	-0.01	-0.04
	(0.02)	(0.03)	(0.03)	(0.02)	(0.03)
2	0.03	-0.02	0.02	0.00	-0.02
	(0.02)	(0.03)	(0.03)	(0.02)	(0.03)
3	0.05^+^	0.01	-0.00	0.01	-0.06
	(0.03)	(0.04)	(0.04)	(0.02)	(0.05)
4 or more	0.01	-0.02	-0.04	0.04	0.04
	(0.04)	(0.06)	(0.05)	(0.03)	(0.06)
**Elderly in household**					
no	(ref.)	(ref.)	(ref.)	(ref.)	(ref.)
yes	0.02	-0.00	-0.02	-0.02	0.01
	(0.02)	(0.04)	(0.03)	(0.02)	(0.03)
Constant	0.02	0.33*	0.84***	0.86***	0.58***
	(0.06)	(0.15)	(0.08)	(0.05)	(0.11)
Location fixed effects	Yes	Yes	Yes	Yes	Yes
*N*	1426	1666	1481	1666	1301
*R* ^2^	0.23	0.14	0.10	0.06	0.15

OLS regression of the dependent variable capturing vaccination take-up by survey wave (see model header: binary indicator of at least one shot, models 1–5; at least one booster shot, model 6) on sociodemographic characteristics of respondents. Location-fixed effects are included. Location cluster-robust standard errors are presented in parentheses. The centered variance inflation factor is between 1.75 (model 1) and 1.70 (model 3) on average, with no single VIF larger than the cut-off of 5 for problematic values.

^+^ (*p* < 0.10),

* (*p* < 0.05),

** (*p* < 0.01),

*** (*p* < 0.001).

## Results and interpretation

### Descriptive evidence on COVID-19 affectedness, vaccination intentions, and behavior

We start with presenting summary statistics for the variables used in our analysis (as of December 2021) in Table 1. The top of Table 1 describes the survey participants, and (given random sampling) the underlying population (see discussion in Methods). The bottom of Table 1 presents the distributions of core dependent and independent variables pertaining to vaccination, health behavior, and perceptions, which are discussed in detail below.

**Fig 2:**
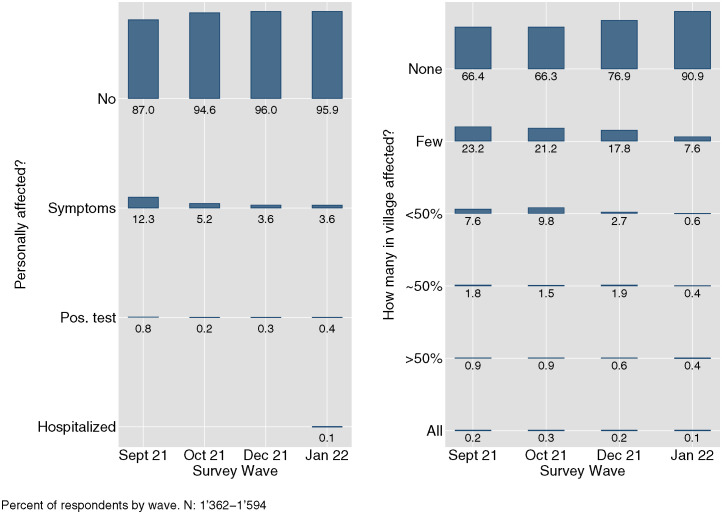
Self-reported affectedness and perceived village-level affectedness with COVID-19 infections by survey wave.

Turning to the results, this section first presents descriptive evidence concerning self-reported COVID-19 affectedness. As depicted in Fig 2, the majority of the survey respondents indicated minimal personal COVID-19 affectedness and perceived low levels of COVID-19 prevalence within their villages. Notably, during the peak of the national-level case numbers observed in summer 2021, coinciding with our phone survey wave conducted in September 2021, a substantial number of respondents (87%) reported experiencing “no symptoms”, while 67% perceived their village as “COVID-19-free”. Instances of hospitalization were extremely rare. Subsequent survey waves revealed a further decline in these figures, indicative of even lower (perceived) COVID-19 prevalence. These findings underscore the notably limited burden of COVID-19 experienced by the remote, rural areas included in our study throughout the duration of the pandemic.

**Fig 3:**
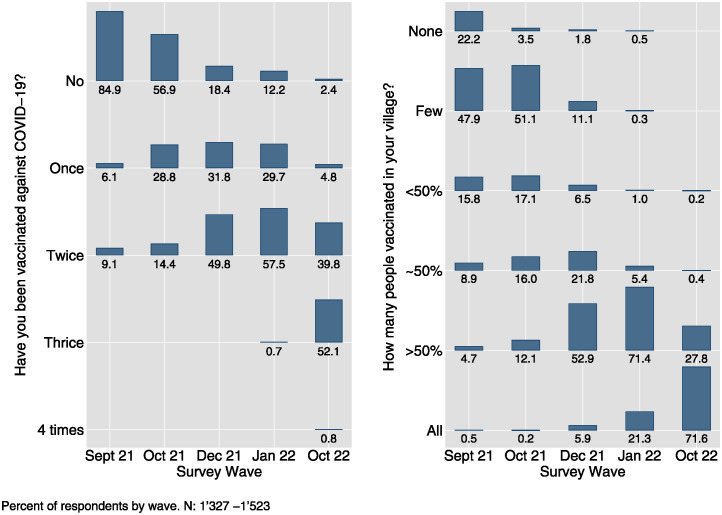
Self-reported COVID-19 vaccinations (left) and perceived village-level vaccination rates (right) by survey wave.

**Fig 4:**
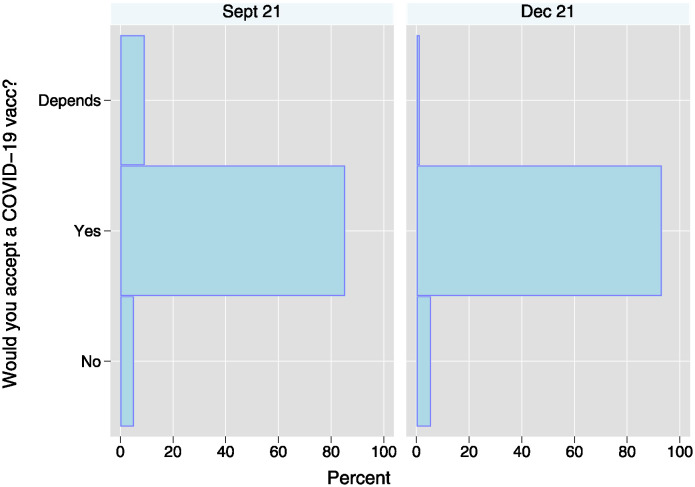
Self-reported vaccination intentions among those reporting to not have received at least one shot. *N*_*Sept*21_: 1134; *N*_*Dec*21_: 234.

At the same time, respondents reported a pronounced willingness to receive the COVID-19 vaccine from the outset of its availability in the country. As illustrated in the left panel of Fig 3, by September 2021, a mere 15% of the population had received at least one vaccine shot. However, as reported in the left panel of Fig 4, there was a considerable inclination towards vaccination, with 85% of respondents who had not yet received the vaccine expressing an unequivocal willingness to do so. This trend persisted in December 2021, as demonstrated in the right panel of Fig 4, despite the widespread availability of vaccines and a reduction in the proportion of unvaccinated respondents to only 14%. Among the study cohort, self-reported willingness to receive the vaccine was remarkably high, hovering at around 93%. With the availability of vaccines, there was a rapid and comprehensive surge in vaccination rates, as evidenced by both self-reported (see left panel of Fig 3) and perceived village-level vaccination rates (right panel), culminating in near-universal vaccination coverage by October 2022.

**Fig 5:**
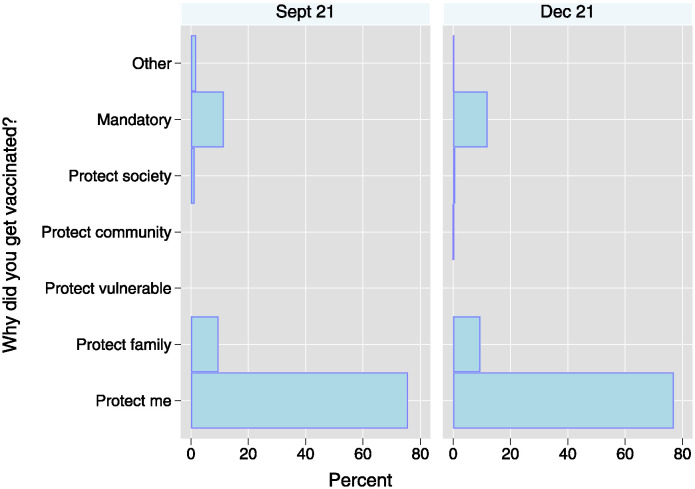
Reasons for getting vaccinated. *N*_*Sept*21_=218; *N*_*Dec*21_=987.

This phenomenon may be attributed to the sustained high motivation among respondents across consecutive survey waves to receive the vaccine. As depicted in Fig 5, the predominant motivation cited by vaccine recipients was personal protection, accounting for 76% to 77% of responses. Additionally, motivations related to safeguarding family members (9%) and a perceived sense of obligation to undergo vaccination (11–12%) were also noted. Importantly, the frequencies of reported reasons remained largely consistent across different waves, indicating a stable pattern of motivation among vaccine recipients over time.

This finding aligns with the prevailing sentiment among respondents who overwhelmingly expressed strong or tentative agreement that COVID-19 was a serious threat to the health of their family and to the country, as well as to the economic well-being of their household and the country, both in September and December 2021. Furthermore, there was a widespread perception of a very high relative risk from COVID-19 both for respondents personally, as well as for their village and the country, with approximately 90% and 70% of respondents perceiving COVID-19 as a threat as significant as or more significant than tuberculosis or monsoon flooding, respectively. Consequently, a substantial majority of the respondents, above 80%, expressed support for the implementation of a mandatory vaccination policy for the general population, including vulnerable occupational groups and populations. Section 1.1 of S1 Appendix presents the respective figures in detail.

### Health belief model for vaccination uptake

We now turn to the examination of the HBM constructs and their influence on the vaccination uptake of our survey respondents. As shown in Table 2, the overall model performs poorly in explaining vaccination uptake, with the exception of the ‘availability’ component –- which is strongly related to both receiving one dose of the vaccine (model 1) or the full set of vaccinations (model 2). Taking the ‘availability’ component out of the model decreases, despite location-fixed effects being included in the analysis, the *R*^2^ from 86% to 14% (models 1 and 3) and from 37% to 18% (models 2 and 4). This underscores the substantial influence of availability for vaccination uptake, and particularly regarding the first dose. Within the framework of the HBM, the other components exhibit either non-significant or weak associations with the uptake of a single dose (models 1 and 3) or being fully vaccinated (models 2 and 4). Significant at a conventional level of 5% is a correlation between personal health satisfaction and vaccination uptake in models 3 and 4, but it becomes insignificant once vaccine availability is included as a regressor (models 1 and 2). This suggests a potentially confounding relationship between health satisfaction and availability—which warrants a cautious interpretation of this finding. To bolster the robustness of our analysis, we also present coefficient estimates derived from logistic regressions in Table 1 in S1 Appendix and thoroughly discuss results using this alternative modeling strategy there. To summarize, and in accordance with the results presented in Table 2, across all models, variables such as health motivation, severity, susceptibility, and social cues seem to be inconsistent predictors of vaccination uptake, while availability consistently emerges as a prime determinant. Furthermore, supplementary findings suggest potential non-linearities in the impact of travel duration, warranting further investigation.

Overall, this supports the notion that motivational factors held limited significance within our context, while availability emerged as the predominant explanatory factor for actual vaccination uptake.

### Sociodemographic correlates of vaccine uptake

As mentioned above, individual-level sociodemographic characteristics play a crucial role in elucidating vaccination intentions via correlates of intent (e.g., education may be correlated with knowledge, and hence willingness) and access (e.g., age—a well-known risk factor considered in numerous vaccination roll-out programs; occupation and socioeconomic status, which pertain to opportunities for formal and informal access to vaccination during periods of scarcity. Consequently, these socioeconomic correlates of vaccination intentions present an interesting avenue to understand the actual vaccine uptake in our remote, rural study setting.

Table 3 presents the correlation between these indicators and vaccine uptake across various time points, spanning from September 2021 to October 2022. Noteworthy findings include a positive association between higher age and vaccine uptake, reaching statistical significance at the 10% or 5% level only for wave 4, in October 2021. Additionally, respondents with certain income sources, particularly government-related occupations, consistently exhibited a notably higher likelihood of vaccine uptake, with an increase in probability of 31 percentage points during wave 3 in September 2021. This increase remained statistically significant at the 0.1% level across all waves except for October 2022 (booster shot). Moreover, also other formal-sector occupational groups such as business owners and employed workers demonstrated higher vaccination rates compared to those engaged in agricultural employment and day laborers, with the difference between business owners and agricultural employment achieving statistical significance at the 5% level for October 2021 and at the 10% level in January 2022. Similarly, differences between employed respondents and agricultural employment (baseline) were particularly pronounced in September 2021, achieving statistical significance at the 5% level. Interestingly, gender did not appear to be related to COVID-19 vaccination uptake in our study. Education emerged as a significant predictor of COVID-19 vaccination early in the pandemic with notable differences observed among respondents with secondary education in September 2021, significant at the 0.1% level, and in October 2021, significant at the 10% level. However, this association waned in subsequent months, i.e., in December 2021 and January 2022, with education even yielding significantly negative differences in October 2022, significant at the 1% level. Religion, for the most part, did not influence vaccination uptake, except concerning booster uptake, where Muslim respondents were less likely to receive the booster by 31 percentage points at that point. Furthermore, the presence of dependents in the household, such as children or elderly individuals, did not exert any discernible effect on vaccination rates. The model also includes location-fixed effects, which are significant in part and align with expectations based on geographic availability arguments.

To further enhance the robustness of the analysis, we supplemented our findings by presenting coefficient estimates derived from logistic regression in Table 1 in S1 Appendix. Through a thorough discussion of the results obtained with this alternative modeling strategy there, we establish their consistency with the pattern presented in Table 3, thereby reinforcing the validity of our approach.

In the analysis, we identified certain sociodemographic variables that exhibit both statistically significant and substantive associations with vaccination uptake. However, these associations did not remain consistent over time. We, therefore, leveraged the panel property of our dataset to take a dynamic perspective.

**Fig 6:**
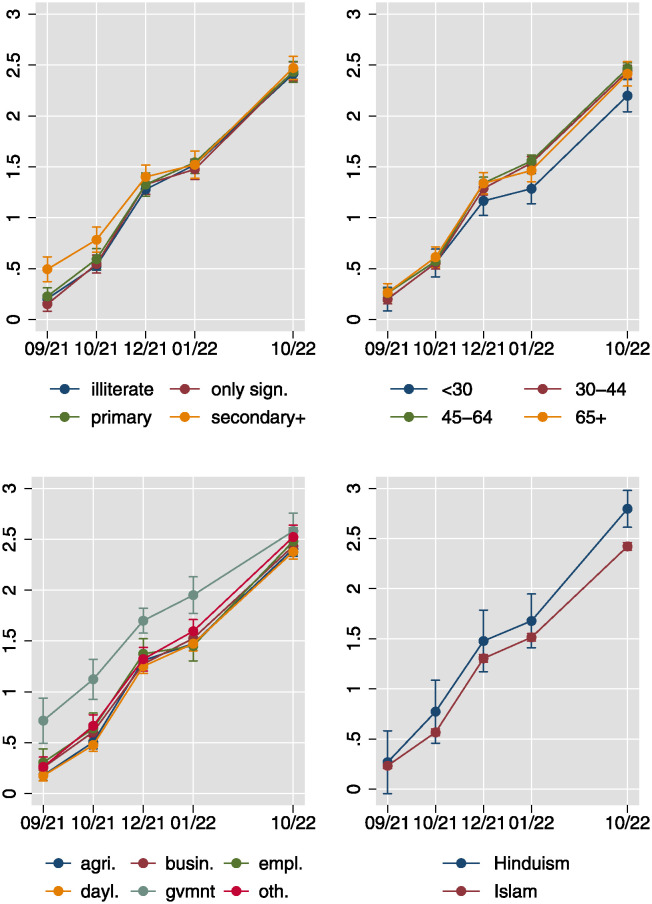
Vaccination uptake over time for four sociodemographic subgroups. Linear predictions of the number of vaccination shots received from location- and survey wave-random effects models with sociodemographics interacted with survey wave. Location-clustered standard errors used.

The results presented in Fig 6 reveal a notable increase in vaccination rates over time, with the average respondent having received approximately 0.25 shots in September 2021 and approximately 2.5 shots in October 2022. Initially, vaccination rates were markedly higher among individuals with high levels of education and those employed in government positions, persisting until December 2021/January 2022, after which they began to converge across different employment/occupational groups. Furthermore, the uptake of the second, and particularly the third vaccination lagged for the youngest age group and for Muslim respondents, relative to older individuals and those identifying as Hindu, respectively. While variation in vaccination rates based on education and government employment may be attributed to differing levels of formal and informal access, such as priority vaccination offerings to government employees like teachers, disparities based on age group and religion are more readily explicable by variations in willingness to uptake the vaccine, possibly influenced by perceptions of threat.

## Discussion

To the best of our knowledge, this is the first study on COVID-19 vaccination intentions and behavior among remote, rural populations in Bangladesh. The study findings, based upon a high-quality panel dataset, indicate that despite the surveyed area experiencing a low burden of COVID-19 affectedness throughout the pandemic, the respondents expressed remarkably high levels of willingness to get vaccinated, corroborating the findings of studies on COVID-19 vaccine acceptance conducted in Bangladesh including rural populations [8, 15, 16]. Additionally, considering that our surveys began around the time when vaccination eligibility was expanded to include nearly all age groups in the country, i.e., in fall 2021, vaccination uptake was significant with almost 50 percent of the respondents reporting receiving one or two doses of the COVID-19 vaccine, contributing to achieving universal vaccination coverage by October 2022. The finding that almost 50 percent of our respondents had been vaccinated by fall 2021 aligns with findings from previous research conducted in rural Bangladesh [8]. The willingness to receive the vaccine was driven primarily by the desire of household heads to safeguard both themselves and their families [8, 37] and remained consistent over time as the respondents perceived COVID-19 to be a threat as significant or even more significant than tuberculosis. This underscores the strong support from over 80 percent of the respondents for implementing a mandatory vaccination policy for the broader population.

While the HBM successfully delineated COVID-19 vaccination intentions as shown in previous studies in Bangladesh and elsewhere [18, 26, 30, 37, 38], its effectiveness in explaining vaccination uptake was found to be limited. Specifically, only the availability component demonstrated an association with vaccine uptake, highlighting that perceived vaccine unavailability and long travel times to reach vaccination centers constituted significant barriers to receiving the vaccine. The study also reveals that certain subgroups, such as well-educated individuals and government employees, were privileged in accessing vaccinations about seven months post-vaccine roll-out, but not eleven months later. This finding was in line with previous research in Bangladesh, showing that these groups were more likely to be aware of the benefits of vaccines and had easier access to them, thus increasing their likelihood of getting vaccinated as soon as the vaccine became available [37, 39, 40]. Similarly, business owners and employed workers were more inclined to get vaccinated compared to individuals engaged in agriculture and day laborers. Respondents aged over 45 were also more likely to have received the COVID-19 vaccine, consistent with previous findings in similar settings [8].

Furthermore, while previous studies noted a higher likelihood of vaccination among females [8, 18], our study found that gender did not influence vaccine uptake. One possible explanation for this finding could be our sampling method, which primarily targeted household heads and thus did not provide a random representation of gender in our dataset. Nonetheless, this result suggests that considering the other factors included in the model, female-headed households did not display any discernible bias in vaccination uptake. Similarly, while religious beliefs have been found to generally predict lower vaccination rates [41], our study showed that religion mostly did not impact vaccination uptake, except in the case of booster shots, where Muslim respondents were significantly less likely to receive the booster. The association between vaccination intentions and actual vaccination behavior entails important policy implications. For instance, if the reported intentions to vaccinate in [13, 17] were followed by vaccination campaigns, it would underscore the importance of ensuring equitable vaccine roll-out, particularly in low-income contexts, and especially in remote areas in these contexts. This is crucial not only for reducing inequality but also due to the potentially high marginal returns of vaccinations in such areas, contributing to global efforts in preventing the spread of COVID-19 and controlling variants.

## Limitations and strengths

Our study focused on a particular area, namely, populations residing along the Jamuna River. As this population is remote and socioeconomically relatively disadvantaged, our findings may not be readily generalizable to the broader Bangladeshi population. Additionally, our self-reported vaccination intentions and behavior data might be subject to social desirability biases. Furthermore, while the study provides a comprehensive examination of sociodemographic factors influencing vaccination intentions and uptake, it does not cover numerous other potential determinants that could shape these outcomes, including trust in government, medical professionals, and healthcare workers, as well as (mis)information disseminated by the media and other outlets regarding COVID-19.

However, this study is one of the few globally to investigate COVID-19 vaccination intentions and uptake utilizing panel data obtained from randomly selected respondents. This approach contrasts with the prevalent use of cross-sectional web-based data derived from unstratified convenience samples. This enhances the external validity of the findings regarding vaccination intentions and uptake among vulnerable and remote populations, which have been understudied. Previous studies, while informative regarding vaccine uptake, have limitations in their applicability to broader populations due to their reliance on cross-sectional online and phone surveys, utilization of marketing companies’ online access panels, or use of un-stratified convenience sampling. These sampling methods reduce the generalizability of findings to the general population, particularly for low-income countries, where internet and cell-phone penetration rates are low, and may overlook urban-rural disparities as rural communities are hard to reach comprehensively with these technologies (e.g. [17, 18, 23]). Potentially, for this reason, existing studies might produce different findings regarding vaccination intentions and uptake. Moreover, studies using social media platforms like Facebook, Messenger, and WhatsApp may result in biased data by inadvertently excluding certain groups, that do not have internet access (e.g., rural residents) or do not feel comfortable navigating online surveys (e.g., low-educated or older individuals), and hence are less likely to participate in such surveys [e.g., 14, 16, 19, 21]. Additionally, this study addresses the challenge of translating vaccination intentions into actual vaccine uptake, a task that has proven difficult for many previous studies [42] since they usually did not rely on panel data and were regularly conducted during ongoing vaccination campaigns and, hence, before supply side constraints were resolved (for rare exceptions, see [8, 9, 23]).

## Conclusion

This study investigates COVID-19 vaccination intentions and uptake among vulnerable and remote populations in Bangladesh, considering sociodemographic as well as health-related and behavioral predictors outlined in the HBM model. The findings revealed a strong willingness among survey respondents to receive the vaccination, consistent with previous research, and this willingness translated into actual vaccine uptake once vaccines became available. Nonetheless, disparities in uptake were noted across socioeconomic groups, likely influenced by factors such as education levels and occupation status, as well as perceptions of COVID-19 risk and prevalence at both individual and village levels. These findings offer valuable insights for health policymakers and healthcare providers, suggesting the need to tailor strategies to enhance COVID-19 vaccine uptake. Notably, the findings suggest that vaccine hesitancy was not a major obstacle, even concerning booster shots. Rather, the primary challenge lay in ensuring equitable vaccine distribution to communities, at least in the rural context of Bangladesh.

## Supporting information

S1 Appendix Supplementary information file. This Supplementary information file contains, in Section 1, additional evidence; in Section 2, the wording for the informed consent statement at the beginning of the questionnaire; in Section 3, the COVID-19-related part of the survey instrument.
(PDF)
