# Supplementary material for: COVID-19 vaccination uptake in remote areas—Evidence from a panel survey in Bangladesh
Source: PLoS One. 2024 Aug 9;19(8):e0305659. doi: 10.1371/journal.pone.0305659 (PMC11315319; doi:10.1371/journal.pone.0305659)
Supplement: S1 Appendix — This Supplementary information file contains, in Section 1, additional evidence; in Section 2, the wording for the informed consent statement at the beginning of the questionnaire; in Section 3, the COVID-19-related part of the survey instrument. (PDF) [file pone.0305659.s001.pdf]

Supplementary Information

‘COVID-19 vaccination uptake in remote areas – Evidence from a panel survey in Bangladesh’

Lukas Rudolph[1] (University of Konstanz), Vally Koubi[2] (ETH Zurich), and Jan Freihardt [3] (ETH Zurich)

PLOS One

June 6, 2024

Contents

1 Additional evidence **2**

1.1 Additional descriptive evidence 2

1.2 Additional regression models 6

2 Additional information on ethics statement **9**

3 Wording of core questionnaire components and link to pre-registered questionnaires **10**

[1]lukas.rudolph@uni-konstanz.de
[2]vassiliki.koubi@ir.gess.ethz.ch
[3]jan.freihardt@ir.gess.ethz.ch

1 Additional evidence

1.1 Additional descriptive evidence

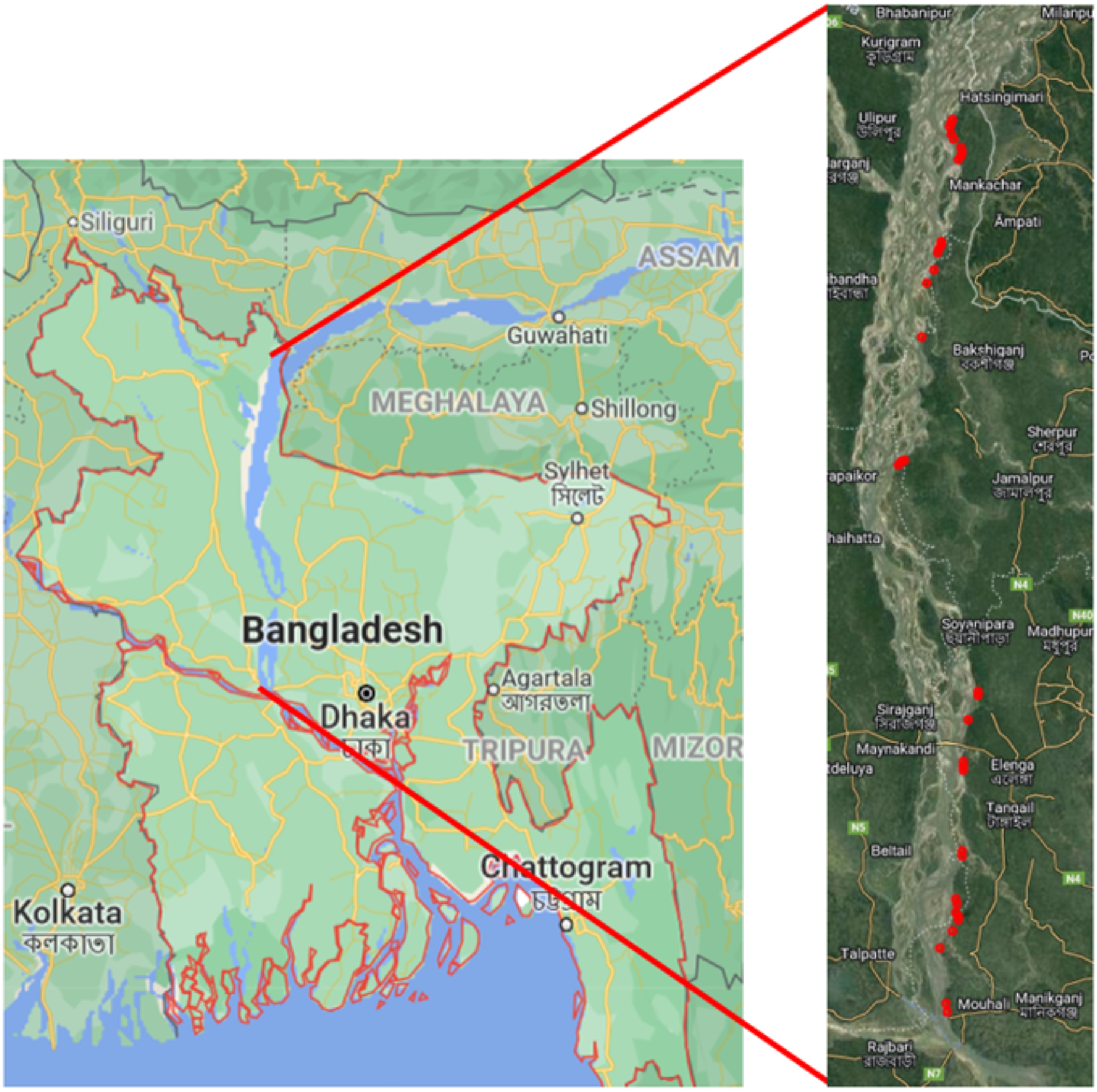

Figure 1. Overview of the 36 study locations. Copyright map: Google. Copyright satellite image: TerraMetrics, 2022.

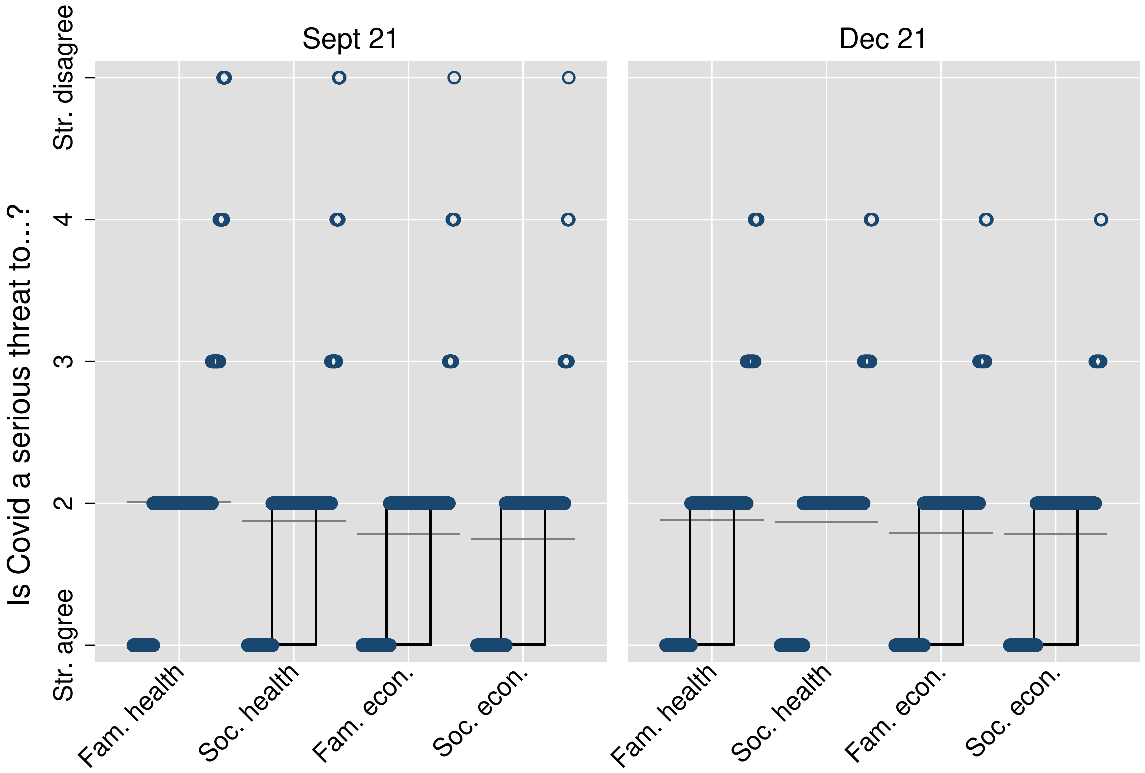

Figure 2. Perceptions of personal and societal COVID-19 threat extent.

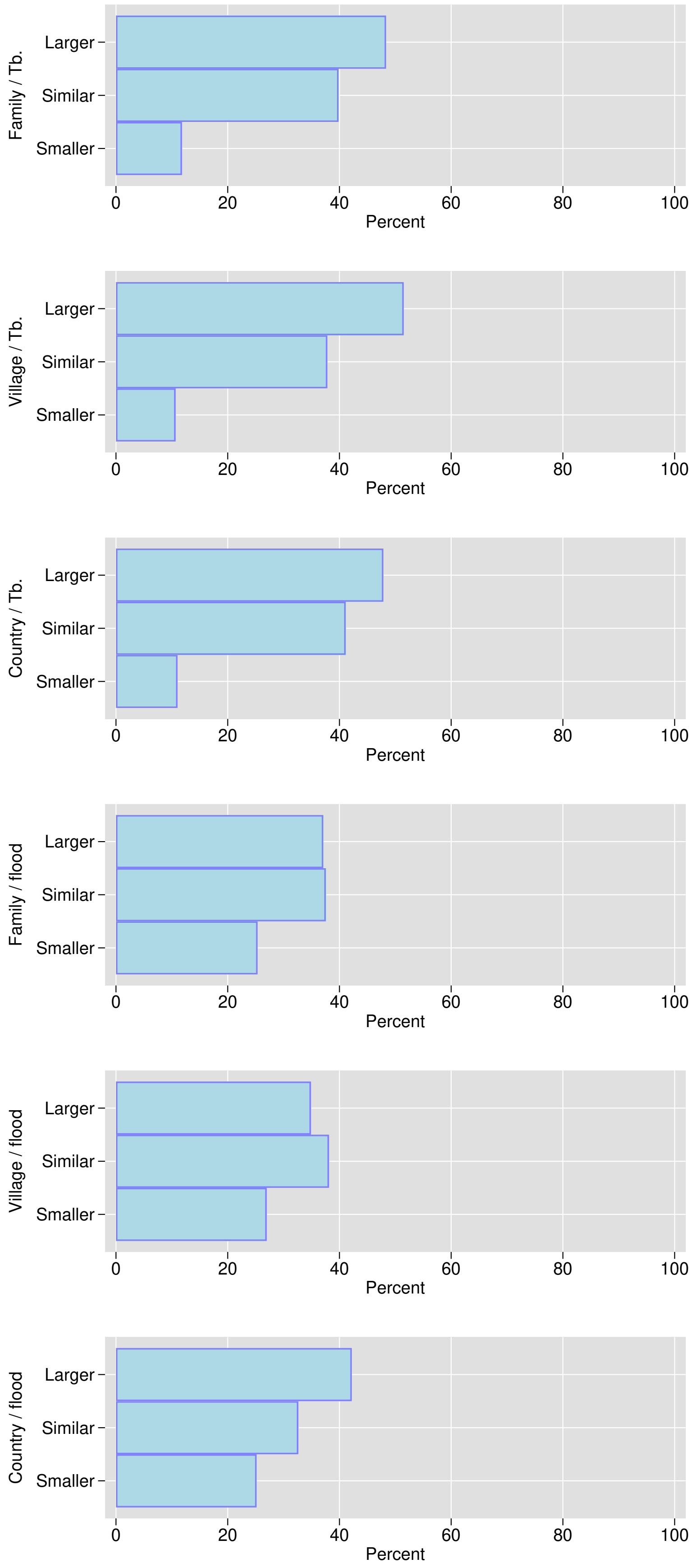

Figure 3. Perception of COVID-19 risk relative to flood/tuberculosis.

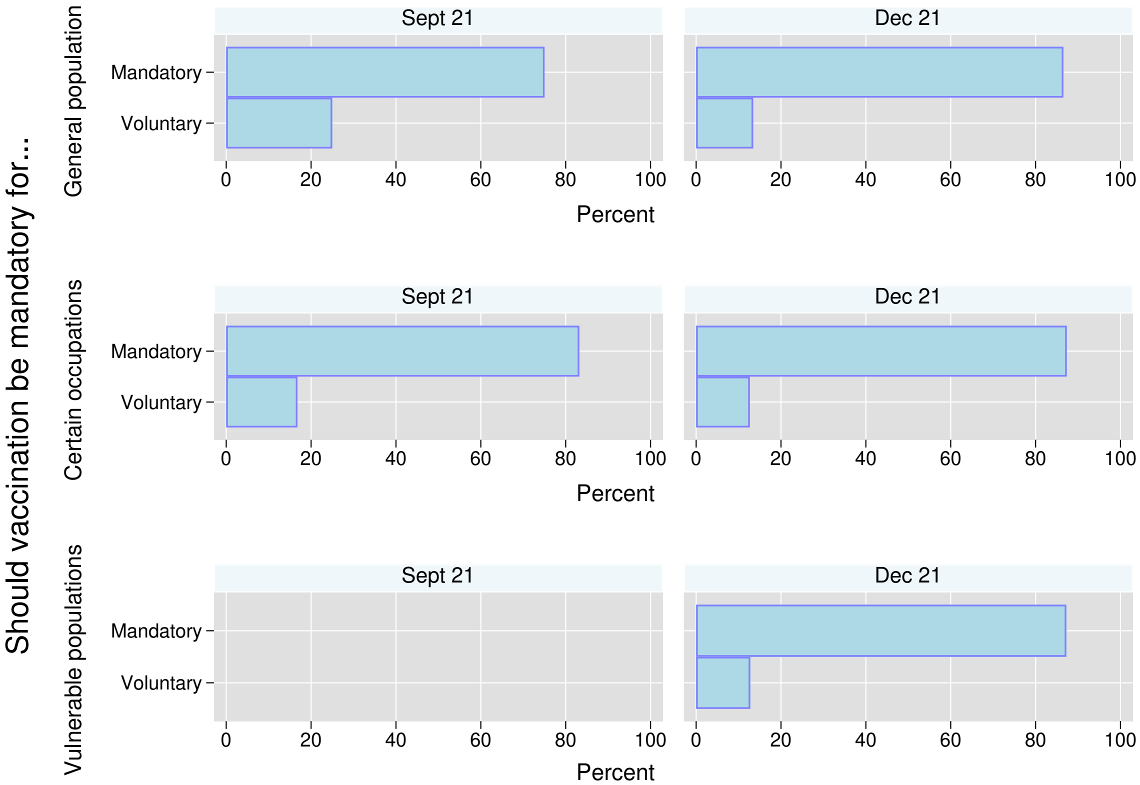

Figure 4. Preferences for mandatory vs. voluntary vaccination policy. Note that the survey item on vulnerable populations (row 3) was not asked in September 2021.

1.2 Additional regression models

This subsection provides logistic regression models as robustness tests for the analyses presented in Tables 2 and 3 of the main text.

Supplementary Information Table 1 corresponds to the analysis presented in Table 2 of the main text in models 1-4. Note that the coefficient for "vaccine not available" cannot be estimated as it predicts failure perfectly, likewise for some of the included village-fixed effects. This also reduces the sample for which this model is estimated. Still, we see that receiving at least one dose of vaccination is not significantly associated to any of the core components of the HBM, besides distance to the district capital. This association is significantly positive, i.e., it has a different sign compared to the main regression table. This is likely due to the change in sample, hinting at non-linearities between distance and vaccination take-up and/or unobserved confounders. Models 2-4 are very much comparable to the estimates presented in Table 2 of the main text. Notably, personal health satisfaction is significantly associated with vaccination take-up in models without fixed effects, as are perceived vaccination rates for receiving the first dose. Given that village-fixed effects also predict failure of vaccination take-up perfectly in some instances, leading to these regressors not being estimatable with logistic regression and the corresponding observations dropping from the estimation sample, therefore changing the inferences we can draw, we also provide results without fixed effects included (models 5-8). Then, the distance coefficient turns positive, though insignificantly estimated. Our inferences for the other coefficients do not change substantively, which indicates robustness of the results presented in the main text.

Supplementary Information Table 2 corresponds to the analysis presented in Table 3 of the main text in models 1-4, with only minor deviations in the results. For example, the number of children is positively, and significantly (at the 1% level for one child, relative to the baseline of no children, and at the 10% level for three children) related to vaccination take-up in September 2021. Otherwise, our results are very much in line with the main text. Given that village-fixed effects also predict failure of vaccination take-up perfectly in some instances, leading to these regressors not being estimatable with logistic regression and the corresponding observations dropping from the estimation sample, therefore changing the inferences we can draw, we also provide results without fixed effects included (model 6-10). Again, the overall pattern of results is remarkably robust, not changing our interpretation substantively.

Table 1. Health belief model for vaccination uptake (cross-sectional, logistic regression).

	(1)<br>1+ vacc Dec 21	(2)<br>2 vacc Dec 21	(3)<br>1+ vacc Dec 21	(4)<br>2 W5 vacc	(5)<br>1+ vacc Dec 21	(6)<br>2 W5 vacc	(7)<br>1+ vacc Dec 21	(8)<br>2 vacc Dec 21
**Health motivation**								
Covid threat to family health	0.50	-0.12	0.13	-0.07	0.33	-0.05	0.07	-0.06
	(0.46)	(0.13)	(0.14)	(0.10)	(0.29)	(0.10)	(0.14)	(0.09)
Personal health satisfaction	0.33	0.22^{+}	0.28*	0.26**	0.18	0.19^{+}	0.33**	0.26**
	(0.30)	(0.12)	(0.11)	(0.10)	(0.19)	(0.11)	(0.11)	(0.09)
Personal Covid affectedness=1	0.57	0.26	0.47	0.29	-0.07	0.16	0.48^{+}	0.08
	(0.79)	(0.22)	(0.32)	(0.21)	(0.54)	(0.19)	(0.26)	(0.18)
**Severity**								
Pandemic ends within 6 months	0.19	0.01	0.11	0.03	0.22	0.03	0.03	0.11
	(0.38)	(0.12)	(0.12)	(0.10)	(0.40)	(0.11)	(0.10)	(0.11)
New wave expected within 6 months	-0.22	-0.15	-0.22^{+}	-0.20^{+}	-0.03	-0.11	-0.17	-0.22^{+}
	(0.31)	(0.14)	(0.14)	(0.11)	(0.27)	(0.13)	(0.13)	(0.12)
**Susceptibility**								
Rather the sinful	0.64	-0.12	-0.07	-0.09	0.69	-0.02	-0.11	-0.05
	(0.66)	(0.32)	(0.36)	(0.26)	(0.61)	(0.29)	(0.29)	(0.21)
Rather the rich	-0.17	-0.28	0.26	-0.17	-0.32	-0.43^{+}	0.30	-0.22
	(0.64)	(0.28)	(0.31)	(0.25)	(0.58)	(0.26)	(0.26)	(0.23)
**Social cues**								
Perceived vaccination rate in village	-0.31	-0.02	0.20*	0.06	-0.32^{+}	-0.01	0.24**	0.10
	(0.24)	(0.10)	(0.09)	(0.09)	(0.18)	(0.09)	(0.07)	(0.08)
Perceived affectedness in village	-0.14	0.14	-0.09	0.10	-0.28	0.16	-0.08	0.15
	(0.26)	(0.15)	(0.12)	(0.13)	(0.27)	(0.15)	(0.10)	(0.12)
**Availability**								
Travel duration most prox. district capital (hrs.)	1.84**	-2.88**			0.09	-0.74**		
	(0.51)	(0.08)			(0.27)	(0.12)		
Vaccine not available (stated)	0.00	0.00			0.00	0.00		
	(.)	(.)			(.)	(.)		
Constant	-5.23**	7.92**	0.23	0.12	2.91^{+}	1.69*	-0.40	-0.92
	(1.77)	(0.94)	(0.67)	(0.62)	(1.62)	(0.75)	(0.56)	(0.61)
Location fixed effects	Yes	Yes	Yes	Yes	No	No	No	No
N	266	690	791	824	694	694	828	828
Pseudo R2	0.09	0.19	0.13	0.14	0.04	0.12	0.04	0.02

Logistic regression of dependent variable indicating vaccination take-up (see model header: binary indicator of at least one dose in models 1 and 3; of two doses, i.e., fully vaccinated, in models 2 and 4) on HBM regressors. Location-fixed effects included in models 1-4, not included in models 5-8. Coefficients with "0.00 (.)" indicate regressors dropped from the analysis as they perfectly predict success/failure. Robust standard errors presented in parentheses. $^{+}(p < 0.10)$, $^{*}(p < 0.05)$, $^{**}(p < 0.01)$, $^{***}(p < 0.001)$.

Table 2. Sociodemographic characteristics and vaccination uptake (cross-sectional models, logistic regression).

	(1) 1+ vacc Sept 21	(2) 1+ vacc Oct 21	(3) 1+ vacc Dec 21	(4) 1+ vacc Jan 22	(5) 1+ boost Oct 22	(6) 1+ vacc Sept 21	(7) 1+ vacc Oct 21	(8) 1+ vacc Dec 21	(9) 1+ vacc Jan 22	(10) 1+ boost Oct 22
**Age**										
30	(ref.)	(ref.)	(ref.)	(ref.)	(ref.)	(ref.)	(ref.)	(ref.)	(ref.)	(ref.)
30-44	0.23	0.12	0.55*	0.56*	0.67*	-0.06	0.10	0.41	0.64**	0.57*
	(0.43)	(0.20)	(0.24)	(0.24)	(0.30)	(0.36)	(0.20)	(0.25)	(0.24)	(0.27)
45-64	0.44	0.22	0.51^{+}	0.57*	0.75**	0.28	0.21	0.37	0.57**	0.78**
	(0.39)	(0.19)	(0.26)	(0.23)	(0.29)	(0.28)	(0.18)	(0.25)	(0.21)	(0.26)
65+	0.68	0.43^{+}	0.56^{+}	0.12	0.76*	0.34	0.43^{+}	0.35	0.17	0.77*
	(0.49)	(0.23)	(0.34)	(0.41)	(0.39)	(0.39)	(0.24)	(0.31)	(0.40)	(0.32)
**Income source**										
agri	(ref.)	(ref.)	(ref.)	(ref.)	(ref.)	(ref.)	(ref.)	(ref.)	(ref.)	(ref.)
business	0.25	0.33*	-0.19	0.47^{+}	-0.08	0.48^{+}	0.38**	-0.31	0.22	-0.05
	(0.23)	(0.15)	(0.20)	(0.25)	(0.23)	(0.27)	(0.14)	(0.21)	(0.20)	(0.21)
employed	0.69*	0.15	-0.02	0.19	0.15	0.81**	0.25	-0.05	0.16	0.18
	(0.32)	(0.23)	(0.35)	(0.40)	(0.24)	(0.28)	(0.19)	(0.29)	(0.36)	(0.27)
daylabor	-0.39	0.13	-0.08	-0.11	-0.09	-0.15	0.07	0.05	-0.07	-0.12
	(0.26)	(0.14)	(0.19)	(0.26)	(0.18)	(0.28)	(0.14)	(0.17)	(0.23)	(0.17)
govnmnt or like	1.98***	1.44***	2.65**	2.55*	0.33	1.55***	1.43***	2.71**	2.38*	0.34
	(0.41)	(0.38)	(0.98)	(1.06)	(0.20)	(0.33)	(0.39)	(0.95)	(1.03)	(0.25)
other	0.26	0.49*	0.07	0.39	0.38^{+}	0.52^{+}	0.55**	0.05	0.26	0.38^{+}
	(0.28)	(0.19)	(0.28)	(0.30)	(0.21)	(0.30)	(0.19)	(0.25)	(0.27)	(0.22)
**Gender**										
Male	(ref.)	(ref.)	(ref.)	(ref.)	(ref.)	(ref.)	(ref.)	(ref.)	(ref.)	(ref.)
Female	-0.28	0.19	0.07	0.22	0.37	-0.19	0.28^{+}	0.01	0.17	0.32
	(0.30)	(0.19)	(0.30)	(0.31)	(0.25)	(0.28)	(0.16)	(0.27)	(0.29)	(0.24)
**Education**										
illiterate	(ref.)	(ref.)	(ref.)	(ref.)	(ref.)	(ref.)	(ref.)	(ref.)	(ref.)	(ref.)
only sign.	-0.26	0.12	-0.08	-0.24	-0.03	-0.00	0.23	0.08	-0.11	0.09
	(0.25)	(0.16)	(0.25)	(0.31)	(0.24)	(0.30)	(0.15)	(0.21)	(0.31)	(0.21)
primary	0.34	0.11	-0.25	-0.57**	-0.02	0.45*	0.13	-0.17	-0.42*	0.12
	(0.27)	(0.21)	(0.24)	(0.19)	(0.20)	(0.23)	(0.20)	(0.21)	(0.19)	(0.19)
secondary+	1.00***	0.40^{+}	-0.01	-0.71*	0.17	1.09***	0.45*	0.12	-0.46	0.33^{+}
	(0.28)	(0.22)	(0.29)	(0.30)	(0.23)	(0.23)	(0.19)	(0.27)	(0.28)	(0.18)
**Religion**										
Hinduism	(ref.)	(ref.)	(ref.)	(ref.)	(ref.)	(ref.)	(ref.)	(ref.)	(ref.)	(ref.)
Islam	-0.73	-0.24	-0.46	-0.63	-2.01**	-0.29	-0.61	-0.63	-1.08	-2.09**
	(0.46)	(0.67)	(0.78)	(1.12)	(0.76)	(0.41)	(0.46)	(0.67)	(1.05)	(0.66)
**Children**										
none	(ref.)	(ref.)	(ref.)	(ref.)	(ref.)	(ref.)	(ref.)	(ref.)	(ref.)	(ref.)
1	0.44*	-(ref.)	-0.15	-0.15	-0.17	0.24	-0.13	-0.14	-0.22	-0.12
	(0.23)	(0.15)	(0.20)	(0.26)	(0.14)	(0.20)	(0.15)	(0.18)	(0.23)	(0.13)
2	0.30	-0.12	0.19	0.09	-0.09	0.28	-0.16	0.13	-0.04	-0.02
	(0.23)	(0.14)	(0.22)	(0.23)	(0.12)	(0.18)	(0.13)	(0.19)	(0.19)	(0.13)
3	0.55^{+}	0.05	-0.02	0.09	-0.27	0.40	-0.11	-0.15	0.05	-0.19
	(0.29)	(0.18)	(0.27)	(0.27)	(0.24)	(0.31)	(0.17)	(0.30)	(0.31)	(0.20)
4+	0.30	-0.10	-0.27	0.62	0.25	0.19	-0.17	-0.39	0.49	0.36
	(0.36)	(0.25)	(0.31)	(0.48)	(0.30)	(0.37)	(0.26)	(0.28)	(0.46)	(0.31)
**Elderly**										
no	(ref.)	(ref.)	(ref.)	(ref.)	(ref.)	(ref.)	(ref.)	(ref.)	(ref.)	(ref.)
yes	0.19	-0.01	-0.16	-0.23	0.04	0.16	0.03	-0.16	-0.33	0.01
	(0.19)	(0.17)	(0.20)	(0.26)	(0.13)	(0.16)	(0.17)	(0.19)	(0.22)	(0.13)
Constant	-3.44***	-0.73	1.83*	2.32*	0.99	-2.37***	0.10	1.82**	2.92**	1.48*
	(0.54)	(0.73)	(0.76)	(1.11)	(0.84)	(0.49)	(0.53)	(0.63)	(1.01)	(0.72)
Location fixed effects	Yes	Yes	Yes	Yes	Yes	No	No	No	No	No
N	1342	1662	1481	1617	1301	1426	1666	1481	1666	1301
Pseudo R2	0.23	0.11	0.10	0.09	0.12	0.08	0.03	0.02	0.03	0.03

Logistic regression of dependent variable capturing vaccination take-up by wave (see model header: binary indicator of at least one shot, models 1-5; at least one booster shot, model 6) on sociodemographic characteristics of respondents. Location-fixed effects included in models 1-5, not included in models 6-10. Location cluster-robust standard errors presented in parentheses. "(ref)" denotes reference category for categorical variables. $^{+}(p < 0.10),^{*} (p < 0.05),^{**} (p < 0.01),^{***} (p < 0.001)$.

2 Additional information on ethics statement

This Supplementary Information section contains the wording for the informed consent statement at the beginning of the questionnaire. Note that ethical approval was obtained after review by the ETH Zurich Ethics Commission (decision EK 2020-N-67).

- For in-person surveys, informed consent was obtained via "[Polite greeting of household head (Assalmo alaikum/Namaskar) depending on religion/village]. I am [interviewer name]. I am a researcher working with a foreign university. I came to this village a few months ago, before the last monsoon, and discussed some questions with you. [Informed consent information] We would like to talk to you again to understand how your life has been since we spoke last time. We do not work with any NGOs or the government. We can thus not provide any help for you or your household. However, our work could inform the Bangladeshi society and decision makers regarding livelihoods along the Jamuna River. Today's interview will last about 45-60 minutes. If you choose to participate, we will pay you 100 Taka at the end of this interview as an appreciation of your time. Of course, you can choose to respond or not respond to any question we ask you. This does not have any negative consequence for you. Similarly, you can stop the interview at any time you want. Your personal information and your responses will be anonymous and no one will ever know that you participated in this survey. From all the interviews taken together, the foreign university will write a detailed report. The report will help all people, the government, and international organizations to better understand how the life of the people in Bangladesh is.
  - QR1: Would you allow that we continue with the survey?
    - a) Yes {have respondents sign/make a cross on the tablet under the statement 'I allow that we proceed with this survey. I understand that I participate on a voluntary basis and can withdraw at any time without giving reasons and without any negative consequences.' Then continue with Q1}
    - b) No {continue with QR2 to end survey}"
- For telephone surveys, informed consent was verbally obtained via "[Informed consent information] I am [interviewer name]. I am a researcher working for a foreign university. We came to your house and talked to you a few weeks ago. I am calling you today to ask a few questions about how your life has developed since we spoke last time. Today's interview will last about 10-15 minutes. If you choose to participate, we will pay you 30 Taka via Bkash at the end of this interview as an appreciation of your time. Of course, you can choose to respond or not respond to any question we ask you. This does not have any negative consequence for you. Similarly, you can stop the interview at any time you want.
  - QR3: Would you allow that we continue with the survey?
    - a) Yes {continue with Q1}
    - b) No {continue with QR2 to end survey}"

3 Wording of core questionnaire components and link to pre-registered questionnaires

In the following, we depict the core components of the questionnaire used for this project. Note that for reasons of brevity, we only include the Covid-19-related questions here.

Note, also, that this research is part of a larger research endeavor focused on researching environmental change and migration. The questionnaire for the pilot (in-person survey) is pre-registered at doi:10.17605/OSF.IO/D4M2Q , for the roll-out of wave 1 (in-person survey) at doi:10.17605/OSF.IO/VXGDB, and the reduced questionnaire for the first phone wave is pre-registered at doi:10.17605/OSF.IO/CQP8R .

Below, we compile the respective parts of the September 2021, October 2021, December 2021, January/February 2022, and October 2022 survey wave questionnaires.

Project on Environmental Migration along Jamuna River, Bangladesh | Phone survey | Wave "Phone 2" / September 2021
ETH Zurich

Corona

Q118: Have you been infected with COVID-19?

1. Yes, tested positive
2. Had symptoms, but was not tested
3. No
99. DK/RA

[If Q118 == yes] **Q119:** Were you hospitalized?

1. Yes (for how long?_______)
2. No
99. DK/RA

Q120: How many people have been infected with COVID-19 in your current village?

1. All households in the village
2. More than half
3. Around half
4. Less than half
5. Only a few
6. None
99. DK/RA

Q121: Have you been vaccinated against COVID-19?

1. Yes, first shot
2. Yes, second shot
3. No
3. DK/RA

[If Q121 == yes] **Q122:** When?

DATE: ________

[If Q121 == yes] **Q123:** Why did you get vaccinated? [MC]

1. to protect myself
2. to protect family
3. to protect individuals who cannot be vaccinated
3. to protect community
4. to protect society
5. It is mandatory
6. other____________
99. DK/RA

[If Q121 == no] **Q124:** Why did you not get vaccinated? [MC]

1. I could not get the vaccine
2. I am healthy and physically fit and so COVID-19 is not a serious risk to myself
3. Others get vaccinated so I do not need to get the vaccine
4. I've already had COVID so I'm protected.
5. I cannot get the vaccine because of health reasons
6. It is a new vaccine and is not sufficiently tested for long-term safety and efficacy, for example infertility issues
7. I am afraid of the side effects
8. I am fearful of vaccines
9. There is no need because COVID-19 is a conspiracy
10. The Covid-19 vaccine is a way for the pharmaceutical industry to make more money
11. I do not trust the health care officials
12. I do not trust the government
13. I do not trust the scientists
14. I do not like the coercive nature of vaccination programmes enacted by the government
15. Allah is protecting me
16. Other___________
99. DK/RA

[If Q124 == "I could not get the vaccine"] **Q125:** Would you accept a COVID-19 vaccination for yourself?

1. Yes
2. Maybe/depends
3. No
4. DK/RA

[If Q125 == yes] **Q126:** Why? [MC]

1. to protect myself
2. to protect family
3. to protect individuals who cannot be vaccinated
4. to protect community
5. to protect society
6. It is mandatory
7. Other___________
99. DK/RA

[If Q125 == maybe] **Q127:** What does it depend on? [MC]

1. When many people are vaccinated
2. When the vaccine becomes mandatory
3. Other__________
99. DK/RA

[If Q125 == no] **Q128:** Why not? [MC]

1. I am healthy and physically fit and so COVID-19 is not a serious risk to myself
2. Others get vaccinated so I do not need to get the vaccine
3. I've already had COVID so I'm protected.
4. I cannot get the vaccine because of health reasons
5. It is a new vaccine and is not sufficiently tested for long-term safety and efficacy, for example infertility issues
6. I am afraid of the side effects
7. I am fearful of vaccines
8. There is no need because COVID-19 is a conspiracy
9. The Covid-19 vaccine is a way for the pharmaceutical industry to make more money
10. I do not trust the health care officials
11. I do not trust the government
12. I do not trust the scientists
13. I do not like the coercive nature of vaccination programmes enacted by the government
14. Allah is protecting me
15. Other___________
99. DK/RA

[If Q121 == no] **Q129:** Have you already registered to receive the Covid vaccination?

1. Yes
2. No
4. DK/RA

Q130: How many people have been vaccinated against COVID-19 in your current village?

1. All households in the village
2. More than half
3. Around half
4. Less than half
5. Only a few
6. None
99. DK/RA

Q131: Should the COVID-19 vaccine be voluntary or mandatory…

a) …for the general population?
b) …for occupational groups that have direct contact with people (e.g. health or transport workers, teachers)?

1. Voluntary
2. Mandatory
3. DK/RA

Q132: Where do you get information on COVID-19? [MC]

1. Newspaper
2. TV
3. Radio
4. Internet
5. Social media
6. Your doctor
7. Health professionals
8. Government
9. NGO
10. Family

11. Friends
12. Neighbors
12. Other___________
99. DK/RA

Q133: How much do you trust the source of the information on COVID-19?

Strongly trust, trust, neither trust nor distrust, distrust, strongly distrust, DK/RA

Q134: How much do you agree with the following statements? (randomize order of statements)

Strongly agree, agree, neither agree nor disagree, disagree, strongly disagree, DK/RA

"Corona is a serious health threat for my family and myself."

"Corona is a serious health threat for the society of Bangladesh."

"Corona is a serious threat for the economic well-being of my household."

"Corona is a serious threat for the national economy."

Corona

Q118: Have you been infected with COVID-19?

1. Yes, tested positive
2. Had symptoms, but was not tested
3. No
99. DK/RA

[If Q118 == yes] **Q119:** Were you hospitalized?

1. Yes (for how long?_______)
2. No
99. DK/RA

Q120: How many people have been infected with COVID-19 in your current village?

1. All households in the village
2. More than half
3. Around half
4. Less than half
5. Only a few
6. None
99. DK/RA

Q121: Have you been vaccinated against COVID-19?

1. Yes, first shot
2. Yes, second shot
3. No
3. DK/RA

[If Q121 == yes] **Q122:** When?

DATE: ________

[If Q121 == no] **Q129:** Have you already registered to receive the Covid vaccination?

1. Yes
2. No
4. DK/RA

Q130: How many people have been vaccinated against COVID-19 in your current village?

1. All households in the village
2. More than half
3. Around half
4. Less than half
5. Only a few
6. None
99. DK/RA

Corona

Q118: Have you been infected with COVID-19?

1. Yes, tested positive
2. Had symptoms, but was not tested
3. No
99. DK/RA

[If Q118 == yes] **Q119:** Were you hospitalized?

1. Yes (for how long?_______)
2. No
99. DK/RA

Q119b: If you think about the people in your family: Has anyone in your family been infected by Corona?

1. Yes
2. No
4. DK/RA

[If Q119b == yes] **Q119c:** How many family members have been infected by Corona?

1. [enter number of people] ______________
4. DK/RA

[If Q119b == yes] **Q119d:** Has anyone from your family died from Corona, or was ill from Corona so badly that you feared for his/her life?

1. Yes
2. No
4. DK/RA

Q120: How many people have been infected with COVID-19 in your current village?

1. All households in the village
2. More than half
3. Around half
4. Less than half
5. Only a few
6. None
99. DK/RA

[If Q120 != none] **Q120b:** If you think about the people in your village: Has anyone died from Corona?

1. Yes
2. No
4. DK/RA

[If Q120b == yes] **Q120c:** How many people in your village have roughly died from Corona?

1. [enter number of people] _____________
4. DK/RA

Q121: Have you been vaccinated against COVID-19?

1. Yes, first shot
2. Yes, second shot
3. No
3. DK/RA

[If Q121 == yes] **Q122:** When?

DATE: _______

[If Q121 == yes] **Q122b:** Which vaccine did you get? [MC]

1. Johnson & Johnson / Jansen
2. Oxford/Astra Zeneca / Covishield
3. BBIBP-CorV (Vero Cells) / Sinopharm
4. Corona-Vac / Sinovac
5. Sputnik V / Gamalea
6. Moderna / mRNA-1273
7. Pfizer/BioNTech / BNT162b2
8. Other: __________
99. DK/RA

[If Q122b != DK/RA] **Q122c:** Are you happy with the vaccine you got or would you have preferred to get a different one?

1. Happy
2. Different one
99. DK/RA

[If Q122c == Different one] **Q122d:** Which vaccine would you have liked to get? [MC]

1. I do not care
3. Johnson & Johnson / Jansen
4. Oxford/Astra Zeneca / Covishield
5. BBIBP-CorV (Vero Cells) / Sinopharm
6. Corona-Vac / Sinovac
7. Sputnik V / Gamalea
8. Moderna / mRNA-1273
9. Pfizer/BioNTech / BNT162b2
10. Other: __________
99. DK/RA

[If Q121 == yes] **Q123:** Why did you get vaccinated? [MC]

1. to protect myself
2. to protect family
3. to protect individuals who cannot be vaccinated
3. to protect community
4. to protect society
5. It is mandatory

6. other___________
99. DK/RA

[If Q121 == no] **Q124:** Why did you not get vaccinated? [MC]

1. I could not get the vaccine
2. I am healthy and physically fit and so COVID-19 is not a serious risk to myself
3. Others get vaccinated so I do not need to get the vaccine
4. I've already had COVID so I'm protected.
5. I cannot get the vaccine because of health reasons
6. It is a new vaccine and is not sufficiently tested for long-term safety and efficacy, for example infertility issues
7. I am afraid of the side effects
8. I am fearful of vaccines
9. There is no need because COVID-19 is a conspiracy
10. The Covid-19 vaccine is a way for the pharmaceutical industry to make more money
11. I do not trust the health care officials
12. I do not trust the government
13. I do not trust the scientists
14. I do not like the coercive nature of vaccination programmes enacted by the government
15. Allah is protecting me
16. Other__________
99. DK/RA

[If Q124 == "I could not get the vaccine"] **Q125:** Would you accept a COVID-19 vaccination for yourself if it becomes available?

1. Yes
2. Maybe/depends
3. No
4. DK/RA

[If Q125 == yes] **Q126:** Why? [MC]

1. to protect myself
2. to protect family
3. to protect individuals who cannot be vaccinated
4. to protect community
5. to protect society
6. It is mandatory
7. Other___________
99. DK/RA

[If Q125 == Yes] **Q122d:** Which vaccine would you like to get? [MC]

1. I do not care
3. Johnson & Johnson / Jansen
4. Oxford/Astra Zeneca / Covishield
5. BBIBP-CorV (Vero Cells) / Sinopharm
6. Corona-Vac / Sinovac
7. Sputnik V / Gamalea
8. Moderna / mRNA-1273
9. Pfizer/BioNTech / BNT162b2
10. Other: __________
99. DK/RA

[If Q125 == maybe] **Q127:** What does it depend on? [MC]

1. When many people are vaccinated
2. When the vaccine becomes mandatory
3. Other__________
99. DK/RA

[If Q125 == no] **Q128:** Why not? [MC]

1. I am healthy and physically fit and so COVID-19 is not a serious risk to myself
2. Others get vaccinated so I do not need to get the vaccine
3. I've already had COVID so I'm protected.
4. I cannot get the vaccine because of health reasons
5. It is a new vaccine and is not sufficiently tested for long-term safety and efficacy, for example infertility issues
6. I am afraid of the side effects
7. I am fearful of vaccines
8. There is no need because COVID-19 is a conspiracy
9. The Covid-19 vaccine is a way for the pharmaceutical industry to make more money
10. I do not trust the health care officials
11. I do not trust the government
12. I do not trust the scientists
13. I do not like the coercive nature of vaccination programmes enacted by the government
14. Allah is protecting me
15. Other___________
99. DK/RA

[If Q121 == no] **Q129:** Have you already registered to receive the Covid vaccination?

1. Yes
2. No
4. DK/RA

Q130: How many people have been vaccinated against COVID-19 in your current village?

1. All households in the village
2. More than half
3. Around half
4. Less than half
5. Only a few
6. None
99. DK/RA

Q131: Should the COVID-19 vaccine be voluntary or mandatory…

c) …for the general population?
d) …for occupational groups that have direct contact with people (e.g. health, transport or restaurant workers, teachers)?
e) …for specific groups (e.g., older people, university students)

1. Voluntary
2. Mandatory
3. DK/RA

Q138: In the next six months, how do you expect the Corona situation to develop? [randomize order of 3 questions]

How likely do you think it is that the pandemic will end within the next 6 months?	☐ Very likely ☐ Likely ☐ Unlikely ☐ Very unlikely ☐ RA
How likely do you think it is that new Corona waves such as in summer of this year will occur within the next 6 months?	☐ Very likely ☐ Likely ☐ Unlikely ☐ Very unlikely ☐ RA
How likely do you think it is that a new Corona lockdown such as in summer of this year will occur within the next 6 months?	☐ Very likely ☐ Likely ☐ Unlikely ☐ Very unlikely ☐ RA

Q139: Have you ever heard of…? [randomize order of 2 options]

… the Omicron-variant of Corona?	☐ Yes ☐ No ☐ RA
… the Delta-variant of Corona?	☐ Yes ☐ No ☐ RA

[randomly choose and ask only one of the following 3-question-blocks – either for the family (a), the village (b), or the national level (c)]

Q134a: How much do you agree with the following statements? (randomize order of statements)

Strongly agree, agree, neither agree nor disagree, disagree, strongly disagree, DK/RA

"Corona is a serious health threat for my family and myself."

"Corona is a serious threat for the economic well-being of my household."

Q140a: In Bangladesh, each year 60.000 people die due to tuberculosis, and 3.6 lakh are hospitalized. Compared to health threats like tuberculosis, is Corona a smaller, a similar, or a larger threat to…

… the health of your family?	☐ Smaller ☐ Similar ☐ Larger ☐ DK ☐ RA

Q141a: Compared to economic threats like a severe monsoon flood, is Corona a smaller, a similar, or a larger risk to…

… the economic situation of your family?	☐ Smaller ☐ Similar ☐ Larger ☐ DK ☐ RA

Q134b: How much do you agree with the following statements? (randomize order of statements)

Strongly agree, agree, neither agree nor disagree, disagree, strongly disagree, DK/RA

"Corona is a serious health threat for my village."

"Corona is a serious threat for the economy of my village."

Q140b: In Bangladesh, each year 60.000 people die due to tuberculosis, and 3.6 lakh are hospitalized. Compared to health threats like tuberculosis, is Corona a smaller, a similar, or a larger threat to…

… the health of the people in your village?	☐ Smaller ☐ Similar ☐ Larger ☐ DK ☐ RA

Q141b: Compared to economic threats like a severe monsoon flood, is Corona a smaller, a similar, or a larger risk to…

… the economic situation of the people in your village?	☐ Smaller ☐ Similar ☐ Larger ☐ DK ☐ RA

Q134c: How much do you agree with the following statements? (randomize order of statements)

Strongly agree, agree, neither agree nor disagree, disagree, strongly disagree, DK/RA

"Corona is a serious health threat for the society of Bangladesh."

"Corona is a serious threat for the national economy."

Q140c: In Bangladesh, each year 60.000 people die due to tuberculosis, and 3.6 lakh are hospitalized. Compared to health threats like tuberculosis, is Corona a smaller, a similar, or a larger threat to…

… the health of the society of Bangladesh?	☐ Smaller ☐ Similar ☐ Larger ☐ DK ☐ RA

Q141c: Compared to economic threats like a severe monsoon flood, is Corona a smaller, a similar, or a larger risk to…

… the economic situation of the society of Bangladesh?	☐ Smaller ☐ Similar ☐ Larger ☐ DK ☐ RA

Q135: What are the main symptoms of Covid-19? [MC]

1. Coughing
2. Fever
3. Running nose
4. Loss of taste or smell
5. Tiredness
6. Sore throat
7. Headache
8. Other: ________

99. DK/RA

Q136: What are good measures to prevent Covid-19 from spreading? [MC]

1. Masks
2. Social distancing
3. Ventilate spaces
4. Vaccination
5. Wash / disinfect hands
6. Cover mouth when sneezing
7. Pray
8. Go to mosque
9. Live faithfully

10. Other: _________
99. DK/RA

Q137: Which parts of the population are most at risk of dying from Covid-19? [MC]

1. Elderly
2. Those with a weak immune system
3. Cancer patients
4. Diabetes patients
5. Those with medical preconditions
6. Cardiovascular patients
7. Sinful people
8. Rich people
9. Poor people
10. Urban residents
11. Other: _________
99. DK/RA

Q142: If you think about who is likely to fall ill from Corona, and who is not likely to fall ill, who will it rather be? Are the infected only [fill from list], mostly [fill from list], both the same, mostly [fill from list] or only [fill from list]? [randomize order of 4 pairs]

Only	Mostly	Both the same	Mostly	Only	DK/RA
... people from cities?				... people from villages?	
... sinful people?				... devout people?	
... rich people?				... poor people?	
... young people?				... old people?	

Corona

Q171: Have you been infected with COVID-19?

1. Yes, tested positive
2. Had symptoms, but was not tested
3. No
99. DK/RA

[If Q171 == yes] **Q172:** Were you hospitalized?

1. Yes (for how long?_______)
2. No
99. DK/RA

Q173: How many people have been infected with COVID-19 in your current village?

1. All households in the village
2. More than half
3. Around half
4. Less than half
5. Only a few
6. None
99. DK/RA

Q174: Have you been vaccinated against COVID-19?

1. Yes, first shot
2. Yes, second shot
3. Yes, booster shot
4. No
99. DK/RA

[If Q174 == yes] **Q175:** When?

DATE: ________

[If Q174 == no] **Q176:** Have you already registered to receive the Covid-19 vaccination?

1. Yes
2. No
4. DK/RA

Q177: How many people have been vaccinated against COVID-19 in your current village?

1. All households in the village
2. More than half
3. Around half
4. Less than half
5. Only a few
6. None
99. DK/RA

Corona

Q199: Have you been vaccinated against COVID-19?

1. Yes, first shot
2. Yes, second shot
3. Yes, first Booster shot (three shots total)
4. Yes, second Booster shot (four shots total)
5. No
6. DK/RA

[If no. shots last time == this time] **Q128:** Why did you not get an additional Corona vaccination? [MC]

1. I could not get the vaccine
1A. I think I already have enough Corona vaccinations
1B. It is not recommended that I get another Corona vaccination
1C. The pandemic is over / there is no need to get vaccinated anymore
2. I am healthy and physically fit and so COVID-19 is not a serious risk to myself
3. Others get vaccinated so I do not need to get the vaccine
4. I've already had COVID so I'm protected.
5. I cannot get the vaccine because of health reasons
6. It is a new vaccine and is not sufficiently tested for long-term safety and efficacy, for example infertility issues
7. I am afraid of the side effects
8. I am fearful of vaccines
9. There is no need because COVID-19 is a conspiracy
10. The Covid-19 vaccine is a way for the pharmaceutical industry to make more money
11. I do not trust the health care officials
12. I do not trust the government
13. I do not trust the scientists
14. I do not like the coercive nature of vaccination programmes enacted by the government
15. Allah is protecting me
16. Other___________
99. DK/RA

[If no. shots last time < this time] **Q200:** When did you receive your last Covid vaccination?

1. A month before September 2021
2. September 2021
3. October 2021
4. November 2021
5. December 2021
6. January 2022
7. February 2022
8. March 2022
9. April 2022
10. May 2022
11. June 2022
12. July 2022

13. August 2022
14. September 2022
15. October 2022
16. November 2022
17. DK/RA

[If no. shots last time < this time] **Q123:** Why did you get vaccinated? [MC]

1. to protect myself
2. to protect family
3. to protect individuals who cannot be vaccinated
3. to protect community
4. to protect society
5. It is mandatory
6. other____________
99. DK/RA

Q177: How many people have been vaccinated against COVID-19 in your current location?

1. All households in the village
2. More than half
3. Around half
4. Less than half
5. Only a few
6. None
99. DK/RA
